# Goal-directed resuscitation therapy in high-risk patients undergoing cardiac surgery (GRICS study): a randomized controlled trial - preliminary results

**DOI:** 10.1186/cc12131

**Published:** 2013-03-19

**Authors:** E Osawa, A Rhodes, J Vincent, J Almeida, J Fukushima, B Pileggi, C Park, L Camara, J Auler Jr, R Chan, M Piccioni, M Lima, F Galas, L Hajjar

**Affiliations:** 1 Heart Institute, São Paulo, Brazil; 2 St George's Healthcare NHS Trust and St George's University of London, UK; 3 Universite Libre de Bruxelles, Brussels, Belgium

## Introduction

A goal-directed resuscitation therapy (GDT) through optimization of cardiac output reduces complications in noncardiac surgeries. We investigated whether the implementation of a GDT protocol in high-risk cardiac surgery with the use of LiDCO Rapid reduces postoperative complications as compared with the standard of care.

## Methods

We performed a prospective and randomized study whereby consecutive patients fulfilling one high-risk criteria (EuroSCORE >5, ejection fraction <50%, recent myocardial infarction, unstable angina or extracardiac arteriopathy) were allocated to GDT or conventional hemodynamic therapy. We excluded patients with endocarditis, previous use of dobutamine, need for IABP, high dose of vasopressors and emergency surgery. The GDT protocol involved hemodynamic resuscitation aimed at a target of a cardiac index >3 l/minute/m2 through a three-step approach: fluid therapy of 250 ml lactated Ringer's solution, dobutamine infusion up to a dose of 20 μg/kg/minute, and red blood cell transfusion to reach a hematocrit level above 28%.

## Results

Twenty patients from the GDT group were compared with 20 control patients. Both groups were comparable concerning baseline characteristics and severity scores, except for a higher prevalence of hypertension and heart failure in the GDT group. Intraoperative data showed no difference regarding length of extracorporeal circulation, fluid balance, transfusion or inotropic requirement. Patients from the GDT group were given more fluids within the first 8 hours as compared with the conventional group (1,250 ml vs. 500 ml, *P <*0.001). GDT patients showed a median ICU stay of 3 days (95% CI: 3 to 4) compared with 5 days in control patients (95% CI: 4 to 7). Moreover, hospital stay was less prolonged in GDT patients (10 days vs. 14 days, *P *= 0.043). Inotropic dependence was lower in the GDT group (29 hours vs. 55 hours, *P *= 0.003) as well as the cumulative dobutamine dosage (8 vs. 19 μg/kg/day, *P *= 0.025). Also, GDT group presented a lower incidence of infections, tachyarrithmias and acute renal failure (RIFLE R) when compared with the control group.

## Conclusion

A hemodynamic therapy tailored to an optimized cardiac output reduced the length of ICU stay, vasoactive drug requirement and postoperative complications.

